# Effects of Home Confinement on the Intensity of Physical Activity during the COVID-19 Outbreak in Team Handball According to Country, Gender, Competition Level, and Playing Position: A Worldwide Study

**DOI:** 10.3390/ijerph18084050

**Published:** 2021-04-12

**Authors:** Souhail Hermassi, El Ghali Bouhafs, Nicola Luigi Bragazzi, Shiro Ichimura, Khaled E. Alsharji, Lawrence D. Hayes, René Schwesig

**Affiliations:** 1 Physical Education Department, College of Education, Qatar University, Doha 2713, Qatar; 2 Department of Sports Science, Martin-Luther-University Halle-Wittenberg, 06120 Halle, Germany; bouhafs.elghali@gmail.com; 3 Department of Health Sciences (DISSAL), Postgraduate School of Public Health, 16132 Genoa, Italy; robertobragazzi@gmail.com; 4 Department of Global Fire Science and Technology, Graduate School of Science and Technology, Tokyo University of Science, Chiba 278-8510, Japan; shiro@rs.tus.ac.jp; 5 Department of Physical Education & Sport, College of Basic Education, Al-Ardiya 92400, Kuwait; khalsharji@gmail.com; 6 School of Health and Life Sciences, University of the West of Scotland, Glasgow G72 0LH, UK; Lawrence.Hayes@uws.ac.uk; 7 Department of Orthopaedic and Trauma Surgery, Martin-Luther-University Halle-Wittenberg, 06120 Halle, Germany; rene.schwesig@uk-halle.de

**Keywords:** COVID-19, home confinement, team handball, physical activity, stress, lockdown

## Abstract

This study investigated effects of home confinement on physical activity (PA) in Team Handball during the COVID-19 outbreak. A total of 1359 handball players participated (age: 23 ± 6 years). Participants from Europe, Western Asia, and North Africa answered an online version of the International Physical Activity Questionnaire (IPAQ) considering “before” and “during” confinement. COVID-19 home confinement has had a negative effect on PA (vigorous, moderate, walking, and overall). The largest decrease was in the sum parameter “all PA” (MET (metabolic equivalent of task)-min/week, η_p_^2^ = 0.903; min/week, η_p_^2^ = 0.861). Daily sitting time increased from 2.7 to 5.0 h per weekday (*p* < 0.001, η_p_^2^ = 0.669). For gender, continent, country, level of handball league, and playing position, no significant differences (group and interaction effects) were observed. The largest change in PA behavior was in walking (minutes per day: η_p_^2^ = 0.755), with males displaying the greatest decrease (from 62 ± 11 to 30 ± 14 min per weekday; *d* = 2.67). In terms of magnitude, difference between genders was greatest for sitting time (difference in *d* = 1.20). In conclusion, while COVID-19 measures were essential to preserve public health, PA was compromised and sedentary behavior increased because of these public health measures regardless of gender, playing position, and competition level.

## 1. Introduction

Since late December 2019, the world seems to have come to a standstill with the Coronavirus disease 2019 (COVID-19) outbreak. In the face of the ongoing pandemic, public health authorities and governments have enforced increasingly restrictive recommendations and escalation measures, including self-isolation, quarantine, and even lockdowns of entire communities and territories [1,2].

These restrictions are necessary to curb infection rates, yet such limitations may compromise normal daily activities, traveling, physical activity (PA), and adherence to exercise as a result of gyms being closed, group gatherings being forbidden, and increased social distancing [1,2]. In addition to the previously mentioned measures, curfews have been implemented in some countries, which limit opportunities for outdoor activities. These measures have demonstrably positive short-term effects on virus transmission but may negatively affect population health, via decreased physical fitness, known to influence immune function. This effect may exert more severe long-term effects associated with immunological and cardiopulmonary functioning, and the ability to cope with infection [2].

The mandated restrictions concerning outdoor activities, including the regular practice of exercise and PA during the COVID-19 outbreak, are leading to reductions in exercise and PA. In fact, even athletes that complete strength and endurance training programs at home have suffered from reduced aerobic capacity [3]. Consequently, this may contribute to anxiety, depression, mental health distress, and common chronic health diseases [1,4,5,6,7]. Indeed, Dönmez et al. [8] indicated that most of the professional football players (66%) had post-traumatic stress disorder symptoms that were caused by social isolation and home-quarantine. These psychological issues exert an influence on training and recovery in athletes [9], which may lead to inferior performance compared to pre-lockdown. Concerning PA, frequency and duration would expectedly decrease in physically active people as a result of an inability to access gyms and health clubs, and reduced need for active transport (i.e., cycling or walking to work) [1,7,10]. Taken together, the pandemic exerts negative patterns on PA within the general population, but the effect on more competitive, and thus highly active individuals, is less known [7]. As a result of this paucity of data, and our previous experience in team handball, we sought to gain an understanding of the COVID-19 restrictions on PA in team handball players [9].

Handball is an Olympic sport played worldwide and at a professional level in many countries, relying on high-intensity intermittent activities with increased demands for muscular strength, explosive strength, speed, agility, reactive agility, power, flexibility, and muscular endurance [11]. Results obtained during gameplay, prior to confinement, have shown that backcourt players cover larger distances and spend less time standing and walking, and together with pivots, have higher in-game heart rates and spend longer durations at higher intensities (>80% maximal heart rate) [12]. In contrast, wing players are faster than other playing positions, and pivots endure more body impact than other players [13]. Furthermore, wings are the shortest, have significantly lower body mass and body mass index (BMI) than other players; pivots are the heaviest, whereas other playing positions do not differ in height [11].

As described previously [14], when athletes, especially those who are well trained, are confined to their homes, their cardiorespiratory and neuromuscular adaptations to various types and extents of PA are likely attenuated or ameliorated. Individual characteristics determine the effects of training modification during quarantine [9,15,16], and subsequently, there have been reductions in some components of fitness to a greater extent than others. Previous studies have reported changes in jumping performance, throwing velocity, maximum muscular strength, and upper and lower limb power during a handball season [11]. Moreover, it is conceivable that a lack of team training and official competitions in team sports may have decreased communication between players and coaching staff, resulting in inadequate training programming [17]. In addition, Sonza et al. [18] indicated that the COVID-19 pandemic influenced training practice and habits in term of frequency, duration, motivation, and period to exercise in Brazil and its main macro-regions. Furthermore, following the German lockdown for containment of the COVID-19 pandemic, highly trained kayakers and canoeists spent less overall time training each week (−28%) with, on average, shorter training sessions (−15%) and less light-to-moderate physical activity outside of training [19]. Additionally, de Albuquerque Freire et al. [20] demonstrated that COVID-19-related restrictions and quarantine had adverse effects on professional soccer players’ Yo-Yo Intermittent Recovery Test performance. Therefore, in the context of COVID-19, public health measures such as isolation, curfews, and lockdowns could have resulted in partial or total reversal of the training adaptations (i.e., ‘detraining’) [11,21].

While it is important to note reductions in PA during the pandemic, these findings are not ubiquitous, and may be dependent upon several factors. For example, Lesser and Nienhuis [15] observed that the change in (∆)PA was dependent upon initial PA levels prior to confinement conditions, and Castañeda-Babarro et al. [22] reported that males reported a greater decrease in vigorous PA than women, although both males and females reduced walking time to a similar extent. Moreover, sedentary time reportedly increased more in males than females, suggesting males are more susceptible to PA reductions and sedentary behavior increases than females during pandemics. Taken together, previous literature suggests pre-pandemic PA and biological sex may mediate the effect of the COVID-19 pandemic on subsequent PA levels.

Considering the profound impact COVID-19 has had on working conditions of athletes, the present study considered handball players worldwide from various leagues, competition levels, and playing positions. The study aimed to rapidly assess how COVID-19 may have affected these groups of players during the ongoing crisis and its major consequences on society. Specifically, we aimed to determine changes since the COVID-19 outbreak and how PA of handball players compared to before the pandemic using questionnaires. Our primary hypothesis was that PA would decrease as a result of the restrictions. A secondary hypothesis was that individuals at a higher competition level and males would have experienced a greater reduced in PA than individuals at a lower competition level and females.

## 2. Materials and Methods

An important criterion for the selection of questionnaires was the proof and evidence of validity and reliability [23,24]. The International Physical Activity Questionnaire Short Form (IPAQ-SF) is a multicounty electronic survey designed to assess changes in PA, previously outlined as valid in this context [1,25,26]. The IPAQ-SF started on 21 May 2020, following testing by the project steering group for 7 days. The IPAQ-SF was disseminated worldwide on 27 June 2020 via six research organizations from Europe, North Africa, Western Asia, and North America. All measures were collected on the same day, to avoid bias, considering the constantly evolving situation of the pandemic.

The IPAQ-SF is available to the public in all language versions. In our study, the IPAQ-SF was administered in English, German, French, Arabic, and Japanese. The survey included 25 questions on gender, demographic information (e.g., age, body mass, and height), competition level, handball league, playing position, playing experience, competitions per years, health status, PA (e.g., vigorous, moderate, and walking activity). All PA- and sitting time-related questions were presented in a differential format, to be answered directly in sequence regarding “before” and “during” confinement conditions [1,25,26]. Each item or question requested two answers, one regarding the period before and the other regarding the period during confinement, and participants were guided to compare the situations [1,25,26]. Once the deadline for admitting surveys had passed, answers were reviewed to remove contradictory responses (checking congruence between data provided by players) or repeated (checking two or more submissions with the same responses in a short period of time), deleting one response from the database. Given the large number of questions included, the present paper focuses on the IPAQ-SF as a brief crisis-oriented tool. Participants signed an informed consent form before completing the survey. The study was conducted according to the Declaration of Helsinki and was approved by the university’s institutional review board of Qatar University (QU-IRB 1350EA-2020) for local participants.

### 2.1. Recruitment and Description of the Sample

In total, 1359 of the 1500 invited participants responded. A total of 85% (1153/1359) participants were recruited in Asia (Table 1), while the highest number of investigated subjects were in Japan (*n* = 758, 56%), Kuwait (*n* = 102, 8%), and Saudi Arabia (*n* = 97, 7%).

The sample of men was markedly larger (*n* = 901, 66%) than the number of recruited women (*n* = 458, 34%; Table 2).

The mean age of all participants was 23 years and ranged from 18 to 60 years. BMI varied from 14.3 to 43.4 kg/m^2^ (Table 2). Except for age (η_p_^2^ = 0.013), all differences between males and females reached the a priori level of significance (height: η_p_^2^ = 0.443; weight: η_p_^2^ = 0.526; BMI: η_p_^2^ = 0.194).

A total of 29% (*n* = 391) participants played in the first league (Table 3), while most (46%) players played in the second league (*n* = 631). Only 25% of players played in the third or fourth league (*n* = 337). Two-thirds of the subjects were male (*n* = 901) and age varied from 18 to 45 years (mean age: 22.8 years; Table 3).

### 2.2. Sample Size Calculation

The sample size was calculated according to the following predictive equation [27]:

(1) $$N=\frac{\left(Z\alpha /2\;2\;p\;q\right)}{\Delta 2}$$

where *N*: number of needed participants; *Z α*/2: two-tailed normal variance for type 1 error; p: change in % from “before” to “during” confinement; *q*: equal to “1 − *p*”; Δ: accuracy; “n” was the number of needed participants; “*Zα*/2” was the two-tailed normal deviate for type 1 error (*Zα*/2 = 1.96 for 95% level of significance); “*q*” was equal to “1 − *p*”; “Δ” was the accuracy (=3%); and “*p*” was the percentage of change in social participation from “before” to “during” confinement period.

Comparable to Ammar et al. [28], the “p” from a recently published study [29] was used. Zhang and Ma [29] examined the immediate effects of the COVID-19 pandemic on mental health and quality of life. Based on these findings, it appeared that 57.8% (*p* = 0.578) of subjects experienced an increase in shared feelings with family members [29]. Consequently, the calculated sample size was *n* = 1041. We recruited our sample size assuming a dropout rate of 40% (*n* = 416). Therefore, we invited 1500 subjects to participate in order to generate a sufficiently large sample size as a precondition for a sufficient calculation and interpretation of effects (meaningful vs. non-meaningful).

### 2.3. Survey Development Promotion and Distribution

A steering group of PhD scientists and academics (in the fields of human sciences, sport science, and computer science) designed the electronic survey at the University of Qatar (where the principal investigator was based). In addition, the survey was subsequently evaluated and amended by handball players, coaches, and International Handball Federation (IHF) handball experts. Thereafter, the survey was reviewed and edited by >35 colleagues and experts before being disseminated via the Google platform (online). Members of the consortium distributed the link to the survey via several methods: e-mail, official faculty pages, ResearchGate™, LinkedIn™, and other social media platforms such as Facebook™, WhatsApp™, and Twitter™. The general public assisted in the dissemination through promotion of the survey within their networks. The selection for the group was deliberate by the research group and the criterion of selection was based to include handball players aged ≥ 18 years of age and in good health (no pain and diagnosis at the time of examination), from different country in the world, different competition leagues, ranking level, amateur or professional, different position, and gender. The exclusion criteria included a positive COVID-19 test or existence of cognitive decline. In total, the uniform resource locator (URL) of the online survey was sent to 1500 potential participants, of which 1359 returned valid questionnaires that were included in the analysis (participation rate of 91%). The description of conditions of lockdown in the countries of the participants is displayed in Table 4.

The survey included an introductory page describing the background and the aims of the survey, the consortium, ethics information for participants, and the option to choose one of five available languages (English, German, French, Arabic, and Japanese). The inclusion criterion was that participants were handball players aged 18 years or older and in good health. No restrictions in terms of playing level, categories, or playing position were made. Exclusion criteria included the existence of any chronic disease or orthopedic condition that might interfere with the participation in the study, and players with cognitive decline. Before completing the survey, individuals voluntarily consented to anonymously participate in this study.

### 2.4. Data Privacy and Consent of Participation

Participants were assured data would be used solely for the research purposed during informed consent gathering. Responses were anonymous in line with Google’s privacy policy (https://policies.google.com/privacy?hl=en, accessed on 27 June 2020). Participants were informed that if they wished to withdraw, responses would not be saved or incorporated into the analyses. Responses were confirmed once participants clicked ‘submit’ [28].

### 2.5. International Physical Activity Questionnaire Short Form (IPAQ-SF)

In line with the IPAQ-SF guidance, the summation from each item (i.e., vigorous intensity, moderate intensity, walking) was used to estimate total PA time per week [23,24]. Weekly PA in MET-min × week^−1^ was calculated by addition of each item multiplied by its respective MET value. The original MT values (original IPAQ) based on the official IPAQ guidelines for young and middle-aged adult (18–65 years old) were used: vigorous PA = 8.0 METs, moderate PA = 4.0 METs, and walking = 3.3 METs. Additionally, we added total PA (sum of vigorous intensity, moderate intensity, and walking activities) as a fourth item and sitting time as a fifth item. The scoring protocol for the IPAQ-SF proposed levels category are:

- Inactive: Those individuals who did not meet the criteria for categories 2 or 3 were considered ‘insufficiently active’;
- Minimally active: The minimum pattern of activity to be classified as ‘sufficiently active’ was when a participant achieved a minimum of at least 600 MET-min/week (category 2);
- HEPA active: ‘HEPA active’ individuals (health enhancing physical activity; a high active category) performed vigorous intensity activities, achieving a minimum of at least 3000 MET-min/week (category 3).

### 2.6. Statistical Analyses

All statistical analyses were performed using SPSS version 25.0 for Windows (SPSS Inc., IBM, Armonk, NY, USA). Following a test of normal distribution (Shapiro-Wilk Test), showing that all variables were not normally distributed (*p* < 0.001), data were analyzed using two-way analyses of variance (ANOVAs). ‘Time’ was considered the within-subject factor, and the between-subject factors were ‘gender’ or ‘handball league,’ depending on the analysis being conducted. Furthermore, we used the variables country and continent as control variable in order to evaluate the level of participation in different countries and on different continents. Subsequently, pairwise effect size was calculated for each parameter by dividing the mean difference of the variable by the pooled standard deviation, which could then be interpreted as small effects (d < 0.5), moderate effects (d ≥ 0.5), and large effects (d > 0.8) [30]. A positive effect size represents an improvement, and a negative value represents a decrement in said variable. Percentage changes were calculated as ([post-confinement value-pre-confinement value]/pre-confinement value) × 100. Differences were considered as meaningful if *p* < 0.05, with partial eta-squared (η_p_^2^) > 0.10, while the effect size (d) was ≥0.8 [31]. Data are reported as means ± standard deviations (SD).

## 3. Results

### 3.1. Physical Activity Depending on Country of Origin

In order to evaluate PA in different countries and continents, we used country and continent as control variables within variance analysis (Table 5).

The time effects for PA parameters (vigorous or moderate physical activities and walking) and sedentary behavior (measured by sitting time) ranged from η_p_^2^ = 0.06 (walking; days for at least 10 min) to η_p_^2^ = 0.67 (vigorous PA; MET-minutes per week; Table 4). The paired effect sizes (d) considering before vs. during confinement varied from d = 0.23 (vigorous PA/days/week) to d = 5.23 (moderate PA/MET-minutes per week). Meaningful continent or nations effects were not observed for one of the investigated PA parameters (Table 5). Confinement impacted participant IPAQ-SF categorization and reduced the mean values from HEPA active to minimally active.

### 3.2. Level of Handball League and Gender Comparison

For all PA variables, no effects of playing level (handball league) were observed (rejection of our primary hypothesis). Time effects for PA variables (vigorous or moderate physical activities and walking or sitting) ranged from η_p_^2^ = 0.13 (walking days/walk for at least 10 min) to η_p_^2^ = 0.49 (vigorous MET-minutes per week). No significant differences (gender effects) between male and female were observed (Table 6). In males (from 2492 ± 654 to 740 ± 500 MET-min/week; d = 3.04) and females (from 2122 ± 721 to 528 ± 384 MET-min/week; d = 2.89), vigorous MET-minutes per week showed the largest effect size. The smallest reduction (η_p_^2^ = 0.13; d_female_ = 1.14, d_male_ = 1.39) over time for both sexes was observed for walking. The largest change in PA parameters was detected for “all physical activity,” whereby the energy expenditure (min/week: η_p_^2^ = 0.52) was significantly decreased during confinement when compared to before confinement (Table 6).

**Figure 1:**
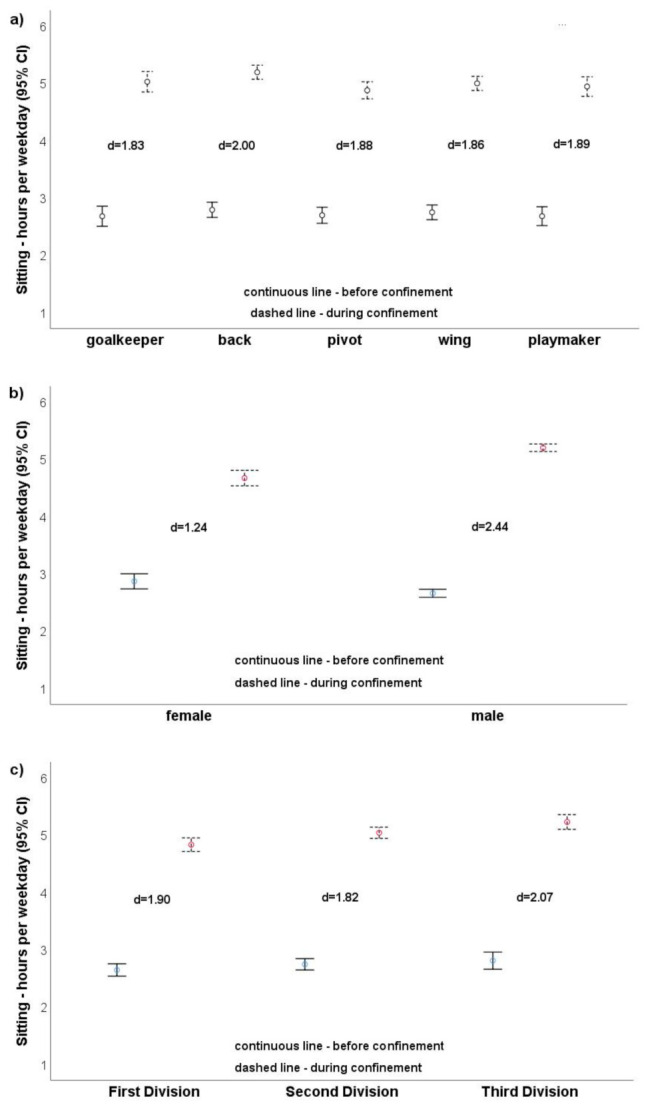
(**a**–**c**). Sitting hours per weekday depending on playing positions (**a**), gender (**b**), and level of handball league (**c**).

**Figure 2:**
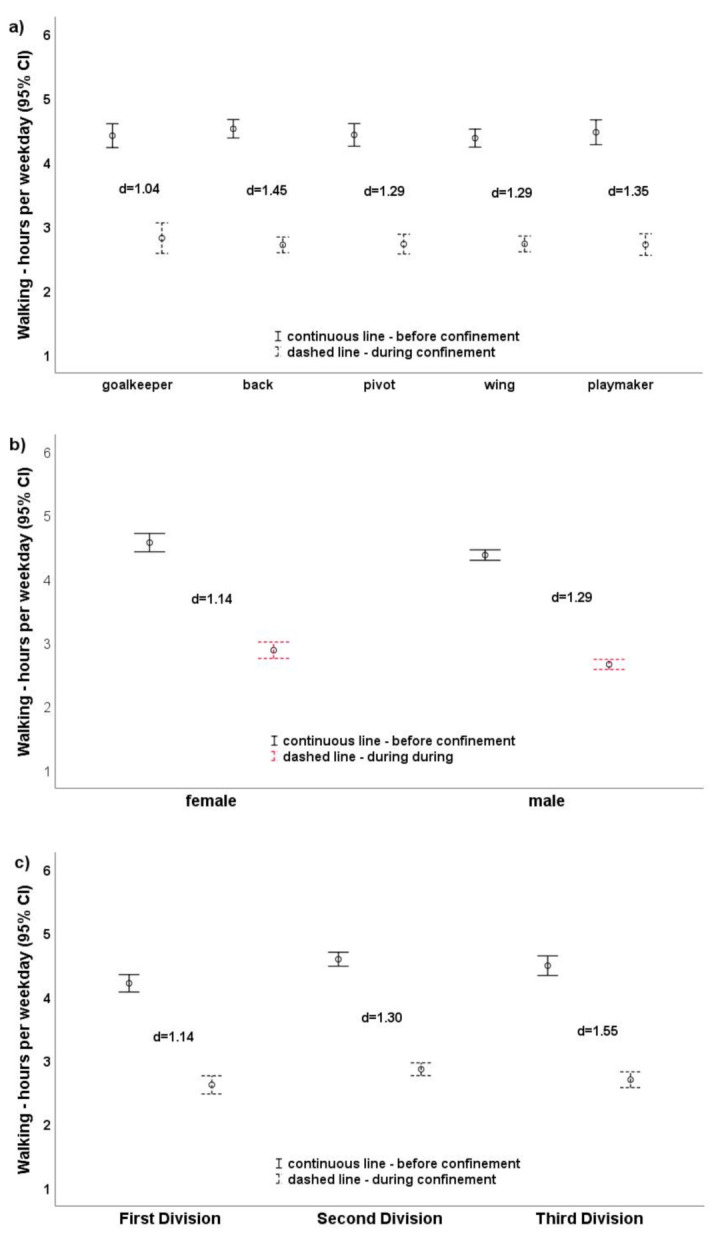
(**a**–**c**). Walking hours per weekday depending on playing positions (**a**), gender (**b**), and level of handball league (**c**).

Playing position (*p* = 0.083, η_p_^2^ = 0.01), gender (*p* = 0.137, η_p_^2^ = 0.00), and level of handball league (*p* = 0.001, η_p_^2^ = 0.01; Figure 1a–c) did not influence sitting time. PA changes were large in all playing positions (Figure 1a and Figure 2a) and were similar in magnitude (sitting: goalkeepers d = 1.83, backs d = 2.00; walking: goalkeepers d = 1.04, backs d = 1.45). Similar results were observed for the influence of level of handball league (Figure 1c and Figure 2c). The sitting effects ranged from d = 1.82 (second division) to d = 2.07 (third division) and the walking effects from 1.14 (first division) to 1.55 (third division). Regarding gender (Figure 1b and Figure 2b), males showed a markedly greater reduction (d = 2F.44, d = 1.39) than females (d = 1.24, d = 1.14).

## 4. Discussion

The results presented here suggest COVID-19 had a negative effect on all intensities of PA in competitive handball players regardless of gender, level of handball league, and playing position. Moreover, increases in sitting time were observed in all participants. Large reductions in PA at medium and vigorous intensity were observed, and this may reflect the subtraction of handball team training. A comprehensive understanding of the implications of these effects is yet to be fully elucidated, but we propose that reduced fitness following confinement is a likely outcome, and an issue coaches and practitioners should be cognizant of.

### 4.1. Main Findings

Home confinement by COVID-19 caused a decrease in the amount of walking per week and an increase in sitting time, which reflects PA in daily life. In addition, the amount of PA at vigorous and moderate intensity, which mostly reflected PA in handball training for handball players, decreased during COVID-19 home confinement. The results indicate that COVID-19-induced home confinement affected not only daily life activities but also PA at moderate and vigorous intensity, which is essential for handball players to maintain and improve their physiological capacities, and therefore, performance as handball players at all levels of performance. The PA of handball players was not affected by continent (5), country (29), gender, level of handball league, and playing position.

### 4.2. Impact upon Daily Life

PA exerts well-documented and measured healthogenic effects, and concomitantly, there is clear evidence linking physical inactivity to non-communicable diseases [32]. Many governmental agencies have developed PA guidelines to improve physical and mental health [33,34,35], emphasizing the importance of PA for public health.

In the present study, the number of walking days for at least 10 min per week was decreased from 4.58 ± 1.57 days to 2.89 ± 1.40 days for women and from 4.38 ± 1.25 days to 2.67 ± 1.21 days for men with a decrease of ~40% in women and men. At the same time, the time of each walk decreased from 56.9 ± 13.0 min to 27.2 ± 12.2 min in women and from 62.1 ± 10.6 min to 29.9 ± 13.5 min in men, representing ~50% in both genders. Therefore, energy expenditure of walking per week decreased from 868 ± 364 MET-min/week to 266 ± 188 MET-min/week for women and from 896 ± 301 MET-min/week to 273 ± 192 MET-min/week in men. It is thought that walking PA is part of the activities of daily life, such as moving around the training facility and at work outside of handball training. Thus, the decrease of walking volume in our subjects may reflect an inactive habitual lifestyle. In addition, sitting time would also be considered an indicator of time is spent in the home. The sitting time on weekdays was increased from 2.87 ± 1.44 h to 4.67 ± 1.47 h in women and from 2.66 ± 1.08 h to 5.20 ± 1.00 h in men. These values corresponded to 1.6 time more sitting time in women and 2.0 more sitting time in men. Previous studies have reported healthy population increased time spent viewing television, social networking, using a smartphone, and playing video games during COVID-19 home confinement [1,16]. Our results demonstrate that even athletes have changed their lifestyle to be less active during this pandemic. Moreover, sitting time increased, which we believe indicates a more sedentary lifestyle.

Comparing walking time per week among athletes versus healthy general populations prior to COVID-19 home confinement, walking time per week was two times higher in our study than in a previous study [1]. However, the difference in walking time per week between our study and the previous study became smaller during COVID-19 home confinement. Weekday sitting time in this study was also 1.6 times and 2 times higher among women and men, respectively, than before COVID-19 home confinement. This increase in sitting time was not influenced by playing position, gender, and level of handball league, suggesting all handball players became more sedentary as a result of COVID-19 measures.

According to the results of an international survey concerning the effects of COVID-19 home confinement on PA in healthy populations, sitting time was increased 1.58 times compared to before COVID-19 home confinement [3]. The degree of increase is smaller than the average value among the women and men found in this study. Thus, the impact of physical inactivity in daily life due to COVID-19 home confinement may be greater in athletes than in the general healthy population. It is thought that the greater negative effect was likely due to athletes being more physically active in daily life before COVID-19 home confinement than the normative healthy population.

### 4.3. Impact of COVID-19 on Handball Training

Handball is characterized by repeated high-intensity actions such as jumping, sprinting, and changes of direction, interspersed by lower intensity periods [11,36]. In addition to the internal loads, body contact with opposition players increases the neuromuscular load and recovery requirements [11].

Thus, it is important for daily handball training to simulate the game at vigorous and moderate intensity. In this study, frequency, time, and metabolic expenditure of moderate to vigorous PA per week was reduced during COVID-19 home confinement. In general, handball players train 2–3 h per session, 4–5 times per week [36]. Furthermore, they play one game a week, depending on the time of year [36]. In this study, we observed that the frequency of PA per week (in time per day) at vigorous intensity decreased 49% in women and 57% in men during COVID-19 home confinement, likely resulting in considerable reductions in fitness. Although we were unable to quantify the reduction in fitness as a result of reduced moderate to vigorous PA, Fikenzer et al. [3] reported the COVID-19 lockdown led to a reduction in aerobic capacity of elite handball players without team training, despite the implementation of a home-based strength and endurance training program. Similarly, Mon-López et al. [9] described COVID-19 affected the training load and recovery process and noted emotional intelligence could predict the change in training variables of top-level soccer players. The recent study of Mon-López et al. [9] reported that handball players reduced training intensity and training volume, which is concordant with the present investigation.

Regarding competition level, Mon-López et al. [9] reported a greater training time reduction in professional handball players than in non-professionals, commensurate with the present study. Skoufas et al. [37], who demonstrated that athletes with a higher competitive level reduced their training volume more than others during the off-season or non-competitive periods due to the higher initial levels of PA, found similar results.

Regarding gender, Mon-López et al. [9] showed a greater reduction in training volume in men than in women during COVID-19, which is in accordance with Giustino et al. [38]. These findings do not agree with those presented here, as we found no gender or gender × time effects. At both measurement times, men tended to show a higher level of activity than the women. However, when gender and competitive level were considered together, the decrease in training volume was greater in professional female players than in professional male players. Mon-López et al. [9] indicated that this result could be biased by the presence of greater number of women in the professional handball category (47%) compared to the number of professional male handball players (38%). In fact, PA levels in professional female handball players before isolation were higher and the reduction greater. In this context, professional female athletes reduced their training volume more during quarantine (76%) than professional males (74%). Recently, one study found that a group of semi-professional male football players reduced hamstring muscle strength following 25 days of home confinement due to the COVID-19 lockdown [39].

In some surveys investigating the impact of COVID-19 on training in elite and semi-elite athletes in South Africa [16] and Italy [38], vigorous and moderate intensity PA decreased during home confinement. In fact, the IOC’s survey has reported that many athletes have been unable to train effectively due to the lockdown caused by COVID-19, and cited this as a major problem for athletes [40]. We observed a large impact of COVID-19 home confinement on moderate and vigorous intensity PA in athletes. Thus, we propose that handball player had insufficient frequency, time, and intensity to sustain or improve physical function and performance during home confinement, and these individuals likely have experienced a large reduction in fitness over this time period.

### 4.4. Reasons of Decreased PA at Moderate and Vigorous Intensity

In a survey of PA during lockdown in the Canadian adult population [15], the time of PA at moderate and vigorous intensity of people with originally high activity (MVPA of 302 ± 186 min per week) was not decreased despite lockdown. Their physical activities at moderate and vigorous intensity consisted of individual outdoor exercise, such as walking, running, and cycling. In contrast, the amount of PA at moderate and vigorous intensity among handball players was significantly decreased in this study. The reason for this conflict in results could be the difference between individual PA for recreation and health promotion and competitive team sports [7]. People undertaking individual PA for recreation and health promotion may be able to continue the same exercise during lockdown by performing outdoor activity such as walking, running, and cycling, whereas handball players and those involved in indoor team sports may experience the greatest deleterious effect of confinement. It may be pragmatic to consider that COVID-19 quarantine has had effects at different levels (physical, physiological, psychological, and emotional) due to a change in the athletes’ daily lives and training habits [5,7,16], and these may not be universal among all athletes.

The mandated restrictions concerning engagement in outdoor activities, including regular practice of exercise and PA in the time of the COVID-19 outbreak, are reducing exercising and increasing sedentary behavior, which can consequently contribute to anxiety, depression, and common chronic health diseases [41]. Concerning mental health, it has been reported that lower levels of physical strength is correlated with higher levels of anxiety and hyperactivity-inattention in both males and females [42]. Interestingly, only strength was associated with anxiety, whereas neither cardiorespiratory fitness nor body composition showed such effects [42]. It seems to be noteworthy that for females, connections between cardiorespiratory fitness and depression did not reach the *p* < 0.05 level [42]. The present study indicates no significant effect of lockdown restrictions on depression.

This study did not consider the geographical or physical exercise or PA context. However, we hypothesize that players in this study would be unable to gather in training facilities for training or matches, and may have undertaken individual training in their own home and/or backyard because of lockdown. These athletes may have found it difficult to ensure exercise intensity to improve and maintain physical fitness and performance at the required level for handball players because individuals training at home and/or in their backyards may be unable to perform the high-intensity intermittent exercises or handball-specific exercises such as the team training and matches.

### 4.5. Implications and Good Practices

Handball players’ lives have been disrupted by the COVID-19 pandemic. There are major psychological repercussions of athletes’ confinement due to lifestyle modification, as they have no reference to this new situation [43]. It is evident that athletes must follow a balanced rhythm of life, appropriate nutritional practices, exercise, and sleeping sufficiently [43]. Thus, a recommendation that can be made from the results of this study is that it is important for handball players to create a handball-specific exercise program to prevent disability after returning to the sport [43]. Training during home confinement will typically be limited to strength, power, and muscle endurance exercises, general physical preparation (e.g., aerobic training on a cycle ergometer), and stretching, among other isolation-limited activities. Acute responses to higher intensities and volumes of exercise can involve a greater risk of illness and impaired immune function [44]. In this context, Toresdahl and Asif [45] advised athletes to follow a conservative approach, limiting training sessions to <60 min and to <80% of maximum effort during this time to prevent COVID-19. Herrera-Valenzuela et al. [46] recommended high-intensity interval training (HIIT) for Olympic sports athletes that can be performed at home, to maintain their physical fitness, cardiorespiratory endurance, and musculoskeletal health. Improving balance may improve strength, power, and speed [47] and enhance subsequent training adaptations [48]. Balance training prior to power (plyometric) training can improve the degree of plyometric training adaptations [47]. Since balance exercises can typically be performed without additional equipment, it may be pragmatic for athletes confined during COVID-19 to emphasize balance training. Pedersen et al. [49] indicated that a prescribed home-based and group-based intervention with training time devoted to strength, jump, and sprint ability, yet regulated to maintain sufficient infection control level, was feasible and effective to preserve strength, jumping, and sprinting abilities of high-level female football players during a ~3-month period of a pandemic-induced lockdown.

### 4.6. Limitations

There were several limitations in this research project. However, one pertinent strength is the large sample size considered from multiple continents, enabled by our multilingual IPAQ-SF dissemination. We accept that this study has the disadvantage of a regional bias in the responses to the survey, despite this survey being open for handball players worldwide and consortium colleagues having distributed the link to the electronic survey via a range of methods. For example, 85% (1153/1359) of the participants were recruited in Asia. Handball players from Japan (*n* = 758; 56% related to the whole sample and 66% related to the Asia sample) were significantly overrepresented, while our aim was to reach many populations of handball players. Likely, nominated local colleagues that distributed the link in Japan were handball experts, who had more access to handball clubs, local federation, and teams. Consequently, the sample cannot be described as representative. The main limitation in this study, however, as with all self-reporting, is the possibility of reporting bias of PA (overestimation of PA), which is common among respondents of a self-reported questionnaire. However, we have asked the same questions in the survey about the two different periods (before and during home confinement by COVID-19). Thus, we speculate that the degree of bias is similar based on the internal consistency of respondents. It is also possible that recall bias was manifest, and respondents were unable to correctly recall the amount of PA before confinement.

Regarding gender comparison, we used two groups with significantly different sample sizes (male: *n* = 901 vs. female: *n* = 458). From a statistical point of view, this is disadvantageous for variance analysis as well as the differences regarding performance levels (professionals, semi-professionals, amateurs), age, body mass, and height. Therefore, results may be biased by having unbalanced groups [50] and no far-reaching conclusions can be drawn. Accordingly, results should be interpreted with caution, due to sample imbalance with three countries being more prominent in the sample. Finally, from a methodological point of view, the implementation of research at the beginning of the Covid-19 pandemic does not reflect the epidemic situation in some countries a few months after the study.

Future study designs could consider more variables in relation to training and recovery conditions (e.g., available space, training machines) and mood (e.g., motivations, private, family situation) of the players. Moreover, an improvement in monitoring systems for training quantity and quality would be desirable to draw conclusions that are based on objectively measured PA. For example, wearable technologies may be able to further elucidate the effects of COVID-19 confinement on PA, but this would require skills in big-data and web technologies. Finally, it would be of interest to examine the effect of PA changes on psychological wellbeing and how the decrease in physical activity translates into the sports level of players and their teams in future investigations.

## 5. Conclusions

To our knowledge, this is the first study concerning handball players during COVID-19 that establishes differences by country, gender, playing position, and sporting level. The confinement period has influenced the days, hours, and intensity of PA. Furthermore, COVID-19 confinement induced a marked decrease in PA of all intensities and reduced the mean categorization of participants from HEPA active to minimally active due to COVID-19 impact. These observations may be used to inform development of PA recommendations during prolonged home confinement and reduced ability to train in a team handball and/or indoors setting. There is a need to translate the findings of this research into future recommendations for home-based exercise intervention in order to improve participants’ PA participation to maintain the HEPA active category in handball players during and after the COVID-19 pandemic. Identification of such sporting populations would allow for better-informed and more targeted mitigation strategies.

## Figures and Tables

**Table 1:** Description of the sample (*n* = 1359) regarding origin and handball activity.

		*n*	%
Continent	Asia	1153	85
	Europe	109	8
	Africa	75	5
	North America	11	1
	Australia	11	1
Level of handball league	First	391	29
	Second	631	46
	Third	274	20
	Fourth	63	5
Playing position	Goalkeeper	204	15
	Back	344	25
	Pivot	249	18
	Wing	360	27
	Playmaker	202	15
Playing experience (years)		10.1 ± 5.49 (1–40)	

Results reported as mean ± standard deviation (range).

**Table 2:** Demographic and anthropometric characteristics of all participants (*n* = 1359).

Gender	Male	*n* = 901		66%
	Female	*n* = 458		34%
		Mean	SD	Range
Age (years)		22.8	6.0	18–60
Body height (m)		1.76	0.10	1.48–2.20
Body mass (kg)		78.1	14.9	44–133
Body mass index (kg/m^2^)		25.0	3.8	14.3–43.4

Results reported as mean ± standard deviation (SD) and range (minimum–maximum).

**Table 3:** Description of the male and female players according to age, competition level, and playing position.

	Age	Competition Level	Playing Position
Male players *n* = 901	18–35 years old *n* = 845	First league *n* = 267	Goalkeeper (*n* = 39)
			Back (*n* = 65)
			Wing (*n* = 68)
			Playmaker (*n* = 43)
			Pivot (*n* = 52)
		Second league *n* = 338	Goalkeeper (*n* = 47)
			Back (*n* = 84)
			Wing (*n* = 90)
			Playmaker (*n* = 59)
			Pivot (*n* = 58)
		Third or lower league *n* = 240	Goalkeeper (*n* = 43)
			Back (*n* = 59)
			Wing (*n* = 71)
			Playmaker (*n* = 29)
			Pivot (*n* = 38)
	36–60 years old *n* = 56	First league *n* = 31	Goalkeeper (*n* = 5)
			Back (*n* = 6)
			Wing (*n* = 6)
			Playmaker (*n* = 5)
			Pivot (*n* = 9)
		Second league *n* = 9	Goalkeeper (*n* = 1)
			Back (*n* = 1)
			Wing (*n* = 2)
			Playmaker (*n* = 4)
			Pivot (*n* = 1)
		Third or lower league *n* = 16	Goalkeeper (*n* = 2)
			Back (*n* = 3)
			Wing (*n* = 3)
			Playmaker (*n* = 3)
			Pivot (*n* = 5)
female players *n* = 458	18–35 years old *n* = 450	First league *n* = 91	Goalkeeper (*n* = 11)
			Back (*n* = 26)
			Wing (*n* = 24)
			Playmaker (*n* = 12)
			Pivot (*n* = 18)
		Second league *n* = 279	Goalkeeper (*n* = 46)
			Back (*n* = 83)
			Wing (*n* = 67)
			Playmaker (*n* = 28)
			Pivot (*n* = 55)
		Third or lower league *n* = 80	Goalkeeper (*n* = 9)
			Back (*n* = 16)
			Wing (*n* = 27)
			Playmaker (*n* = 19)
			Pivot (*n* = 9)
	36–60 years old *n* = 8	First league *n* = 2	Goalkeeper (*n* = 0)
			Back (*n* = 1)
			Wing (*n* = 0)
			Playmaker (*n* = 0)
			Pivot (*n* = 1)
		Second league *n* = 5	Goalkeeper (*n* = 1)
			Back (*n* = 0)
			Wing (*n* = 2)
			Playmaker (*n* = 0)
			Pivot (*n* = 2)
		Third or lower league *n* = 1	Goalkeeper (*n* = 0)
			Back (*n* = 0)
			Wing (*n* = 0)
			Playmaker (*n* = 0)
			Pivot (*n* = 1)

**Table 4:** Description of the conditions of lockdown in the different countries of the participants during the first lockdown.

COVID-19 Pandemic Lockdowns						
*n* (%Participants)	Country	Start Date (DD/MM/YYYY)	End Date (DD/MM/YYYY)	Length #	Level	Conditions of the Lockdown
1 (0.1)	Afghanistan	23.02.20	06.04.20	34	National	The Afghanistan National Olympic Committee announced that all sport events were canceled after 14 March, including a Buzkashi league tournament that was being held in Kabul.
27 (2.0)	Algeria	23.3.20	14.05.20	52	National	The Algerian government enforced a general lockdown.
12 (0.9)	Australia	20.03.20	26.11.20	n/a	National	Social distancing rules were imposed on 21 March, and state governments started to close ‘non-essential’ services.
18 (1.3)	Bahrain	25.02.20	9.04.20	33	National	The executive committee of the Bahraini government announced the closure of all non-essential commercial enterprises from 26 March onward. Exceptions to this rule included supermarkets, banks, bakeries, and healthcare facilities. The closure took effect at 7 pm on 26 March and lasted until 7 pm on 9 April.
3 (0.2)	Chile	15.03.20	19.08.20	138	National	Complete lockdown was extended for the entire area of Greater Santiago, some nearby communities, and also for Iquique and Alto Hospicio cities.
1 (0.1)	France	17.03.20	11.05.20	55	National	Essential journeys included shopping for food, travelling to and from work, accessing healthcare, and exercising within 1 km of the home for up to 1 h.
8 (0.6)	Germany	23.03.20	20.04.20	28	National	German states mandated school and kindergarten closures, postponed academic semesters, and prohibited visits to nursing homes to protect the elderly.
5 (0.4)	India	25.03.20	07.06.20	74	National	Complete lockdown of 82 districts in 22 states and Union Territories of the country where confirmed cases have been reported until 31 March.
2 (0.1)	Iran	14.03.20	20.04.20	37	National	Iranian security forces began implementing a nationwide lockdown.
61 (4.5)	Iraq	22.03.20	11.04.20	20	National	Iraq imposed a total nationwide lockdown until March 28 to fight the spread of COVID-19.
2 (0.1)	Italy	09.03.20	18.05.20	70	National	Italy restricted the movement of the population except where necessary, i.e., for work and health circumstances.
9 (0.7)	Jordan	18.03.20	30.04.20	43	National	The country announced on March 20 a nationwide shutdown that closed shops and prohibited the movement of people.
758 (56)	Japan	7.04.20	31.05.20	68	National	The Japanese government declared a state of emergency for seven prefectures on 7 April. The state of emergency expanded to all prefectures on 16 April and was lifted on 31 May. The government encouraged citizens to avoid going out unless necessary. Elementary, junior, and high schools were closed from 2 March to 31 May.
102 (7.5)	Kuwait	10.05.20	31.05.20	21	National	Kuwait on 20 April expanded a nationwide curfew to 16 h a day, from 4 p.m to 8 a.m, and extended a suspension of work in the public sector, including government ministries, until 31 May.
11 (0.8)	Morocco	19.03.20	10.06.20	83	National	Morocco took exceptional measures that limited the movement of citizens by requiring that any movement out of their home be allowed only after obtaining an official mobility document issued by the officials in the cases identified.
1 (0.1)	Poland	13.03.20	11.04.20	29	National	The government introduced a swathe of closures to keep Poland safe as the coronavirus engulfs Europe.
5 (0.4)	Qatar	11.03.200	15.06.20	278	Industrial park	Partial opening
4 (0.3)	Oman	10.04.20	29.05.20	29	National and City	On 10 April, the entire governorate was put under lockdown until 22 April, which was extended twice, with the lockdown being lifted on 29 May. Starting from 13 June until 3 July, lockdowns were imposed in the governorate of Dhofar, the wilayat of Masirah, the wilayat of Duqm, and the areas of Jebel Akhdar and Jebel Shams.
37 (2.7)	Romania	25.03.20	12.05.20	48	National	The government implemented a nationwide lockdown until further notice, banning individuals from leaving their homes for nonessential reasons and closing all businesses except for those selling food or pharmaceutical products and those providing veterinary services.
97 (7.1)	Saudi Arabia	29.03.20	21.06.20	260	City	Saudi Arabia on Sunday (29 March) decided to impose a lockdown on a fourth city as the Gulf monarchy struggled to contain the Covid-19 (coronavirus) outbreak.
76 (5.6)	Singapore	07.04.20	01.06.20	55	National	General prohibition of mass movements and gatherings across the country, including religious, sports, social, and cultural activities.
53 (3.9)	Spain	14.03.20	09.05.20	56	National	The government warned citizens they faced a prolonged period of social and movement restrictions as officials struck a downbeat tone.
4 (0.3)	Sweden	31.01.20	01.06.20	n/a	National	The Swedish crisis management is built on a principle of responsibility, which means that the organization who is responsible for an area of activity under normal circumstances is also responsible for that area of activity during a crisis.
4 (0.3)	Thailand	26.03.20	30.09.20	125	National	All commercial international flights were suspended from 4 April, and lockdown measures were implemented in varying degrees throughout the country.
3 (0.2)	Taiwan	26.03.20	03.04.20	29	National	All who arrive into the country must complete a 14-day quarantine upon arrival, except for business travelers from countries determined to be at low or moderate risk, who are subject to five- or seven-day quarantines and must undergo a COVID-19 test.
29 (2.1)	Tunisia	22.03.20	19.04.20	28	National	General lockdown for over a week, preventing people from leaving their homes except to buy necessities or work in certain jobs.
6 (0.4)	United Arab Emirates	17.04.20	17.04.20	22	National	The Emirati government issued a mandatory lockdown and closed commercial centers, malls, and open markets for two weeks, after which they will likely resume. Restaurants were ordered to exclusively offer take-out services.
1 (0.1)	United Kingdom	23.03.20	04.07.20	103	National	The British population was instructed to stay home, except for exercise once a day (such as running, walking, or cycling), shopping for essential items, any medical need, providing care to a vulnerable person, or travelling to work where the work in question was vital and could not be done from home.
7 (0.5)	United States	19.03.20	08.05.20	270	State	Governmental operations and non-essential businesses were to be closed until 30 March.

# = days.

**Table 5:** Comparison of physical activity parameters before and during confinement (control variables: continent and country). Values are given as mean ± SD. Meaningful effects (criteria: *p* < 0.05 and η_p_^2^ > 0.10 and d > 0.8) highlighted in bold. MET = Metabolic Equivalent of Task.

	Handball Players (*n* = 1359)			Variance Analysis/Effects *p* (η_p_^2^)		
	Before	During	d	Time	Continent	Country
**Vigorous physical activities**						
**Days/week**	4.61 ± 1.07	2.33 ± 0.96	0.23	**<0.001** **(0.179)**	<0.001 (0.018)	0.107 (0.002)
**Min/week**	63.8 ± 9.41	34.5 ± 14.9	**2.41**	**<0.001** **(0.147)**	<0.001 (0.016)	0.024 (0.004)
**MET-min/week**	2367 ± 699	669 ± 475	**2.89**	**<0.001** **(0.229)**	<0.001 (0.019)	0.007 (0.005)
**Moderate physical activities**						
**Days/week**	4.25 ± 1.10	2.23 ± 0.88	**2.04**	**<0.001** **(0.138)**	<0.001 (0.028)	<0.001 (0.030)
**Min/week**	61.6 ± 10.2	35.7 ± 14.1	**2.13**	<0.001 (0.092)	<0.001 (0.037)	0.474 (0.000)
**MET-min/week**	1049 ± 331	325 ± 194	**5.23**	**<0.001** **(0.140)**	<0.001 (0.059)	<0.001 (0.016)
**Walking**						
**Days/walk for at least 10 min**	4.45 ± 1.37	2.74 ± 1.28	**1.29**	<0.001 (0.064)	0.418 (0.000)	<0.001 (0.017)
**Minutes per walking days**	60.3 ± 11.7	29.0 ± 13.1	**2.52**	<0.001 (0.090)	<0.001 (0.016)	0.848 (0.000)
**MET-min/week**	887 ± 324	270 ± 191	**2.40**	**<0.001** **(0.136)**	0.052 (0.003)	<0.001 (0.010)
**Sitting**						
**Hours per weekday**	2.73 ± 1.22	5.02 ± 1.21	**1.89**	**<0.001** **(0.125)**	<0.001 (0.026)	<0.001 (0.012)
**All Physical Activity**						
**Days/week**	4.44 ± 0.72	2.43 ± 0.69	**2.85**	**<0.001** **(0.263)**	<0.001 (0.015)	0.636 (0.000)
**Min/week**	186 ± 20.7	99.1 ± 29.6	**3.46**	**<0.001** **(0.221)**	<0.001 (0.043)	0.180 (0.001)
**MET-min/week**	4303 ± 908	1264 ± 647	**3.91**	**<0.001** **(0.354)**	<0.001 (0.038)	0.020 (0.004)

**Table 6:** Comparison of physical activity parameters between male and female before and during confinement (control variable: playing level). Values are given as mean ± SD. Meaningful effects (criteria: *p* < 0.05 and η_p_^2^ > 0.10 and d > 0.8) are highlighted in bold.

	Male (*n* = 901)			Female (*n* = 458)			Variance Analysis/Effects *p* (η_p_^2^)		
	Before	During	d	Before	During	d	Time	Gender	Playing Level
**Vigorous physical activities**									
**Days/week**	4.75 ± 0.97	2.43 ± 0.95	**2.42**	4.33 ± 1.19	2.13 ± 0.95	**2.06**	**<0.001 (0.359)**	<0.001 (0.049)	<0.001 (0.015)
**Min/week**	65.3 ± 9.02	36.9 ± 14.8	**2.39**	60.8 ± 9.43	29.7 ± 13.9	**2.67**	**<0.001 (0.345)**	<0.001 (0.089)	0.005 (0.006)
**MET-min/week**	2492 ± 654	740 ± 500	**3.04**	2122 ± 721	528 ± 384	**2.89**	**<0.001 (0493)**	<0.001 (0.089)	<0.001 (0.014)
**Moderate physical activities**									
**Days/week**	4.29 ± 1.08	2.28 ± 0.89	**2.04**	4.18 ± 1.14	2.12 ± 0.85	**2.07**	**<0.001 (0.225)**	0.001 (0.008)	0.005 (0.006)
**Min/week**	62.9 ± 9.89	35.5 ± 13.3	**2.36**	59.0 ± 10.3	36.0 ± 15.6	**1.78**	**<0.001 (0.245)**	0.001 (0.008)	0.032 (0.003)
**MET-min/week**	1082 ± 332	328 ± 188	**2.90**	986 ± 320	319 ± 205	**2.54**	**<0.001 (0.363)**	<0.001 (0.016)	0.040 (0.003)
**Walking**									
**Days/walk for at least 10 min**	4.38 ± 1.25	2.67 ± 1.21	**1.39**	4.58 ± 1.57	2.89 ± 1.40	**1.14**	**<0.001 (0.131)**	<0.001 (0.009)	0.886 (0.000)
**Minutes per walking days**	62.1 ± 10.6	29.9 ± 13.5	**2.67**	56.9 ± 13.0	27.2 ± 12.2	**2.36**	**<0.001 (0.379)**	<0.001 (0.041)	0.453 (0.000)
**MET-min/week**	896 ± 301	273 ± 192	**2.53**	868 ± 364	266 ± 188	**2.18**	**<0.001 (0.342)**	0.140 (0.002)	0.490 (0.000)
**Sitting**									
**hours per weekday**	2.66 ± 1.08	5.20 ± 1.00		2.87 ± 1.44	4.67 ± 1.47		**<0.001 (0.260)**	0.004 (0.006)	0.003 (0.006)
**All Physical Activity**									
**Days/week**	4.47 ± 0.67	2.46 ± 0.65	**3.05**	4.36 ± 0.81	2.38 ± 0.74	**2.56**	**<0.001 (0.439)**	0.002 (0.007)	0.001 (0.008)
**Min/week**	190 ± 19.8	102 ± 29.2	**3.59**	177 ± 19.4	92.9 ± 29.3	**3.45**	**<0.001 (0.524)**	<0.001 (0.079)	0.044 (0.003)
**MET-min/week**	4470 ± 860	1341 ± 680	**4.06**	3976 ± 911	1113 ± 547	**3.93**	**<0.001 (0.138)**	<0.001 (0.693)	<0.001 (0.012)

## Data Availability

The raw data supporting the conclusions of this article will be made available by the authors, without undue reservation.
